# High T2-weighted signal intensity for risk prediction of sudden cardiac death in hypertrophic cardiomyopathy

**DOI:** 10.1007/s10554-017-1252-6

**Published:** 2017-10-23

**Authors:** D. H. Frank Gommans, G. Etienne Cramer, Jeannette Bakker, Hendrik-Jan Dieker, Michelle Michels, Michael A. Fouraux, Carlo L. M. Marcelis, Freek W. A. Verheugt, Janneke Timmermans, Marc A. Brouwer, Marcel J. M. Kofflard

**Affiliations:** 1 Department of Cardiology, Radboud University Medical Center, Nijmegen, The Netherlands; 2 Department of Radiology, Albert Schweitzer Hospital, Dordrecht, The Netherlands; 3 Department of Cardiology, Erasmus Medical Center, Rotterdam, The Netherlands; 4 Department of Clinical Chemistry, Albert Schweitzer Hospital, Dordrecht, The Netherlands; 5 Department of Clinical Genetics, Radboud University Medical Center, Nijmegen, The Netherlands; 6 Department of Cardiology, Albert Schweitzer Hospital, Dordrecht, The Netherlands; 7 Department of Cardiology 616, Radboud University Medical Center, Geert Grooteplein 10, P.O. Box 9101, 6525 GA Nijmegen, The Netherlands

**Keywords:** Hypertrophic cardiomyopathy, Cardiovascular magnetic resonance imaging, Sudden cardiac death

## Abstract

In search of improved risk stratification in hypertrophic cardiomyopathy (HCM), CMR imaging has been implicated as a potential tool for prediction of sudden cardiac death (SCD). In follow-up of the promising results with extensive late gadolinium enhancement (LGE), high signal-intensity on T2-weighted imaging (HighT2) has become subject of interest given its association with markers of adverse disease progression, such as LGE, elevated troponin and non-sustained ventricular tachycardia. In lack of follow-up cohorts, we initiated an exploratory study on the association between HighT2 and the internationally defined risk categories of SCD. In a cohort of 109 HCM patients from a multicenter study on CMR imaging and biomarkers, we estimated the 5-year SCD risk (HCM Risk-SCD model). Patients were categorized as low (< 4%), intermediate (≥ 4–<6%) or high (≥ 6%) risk. In addition, risk categorization according to the ACC/AHA guidelines was performed. HighT2 was present in 27% (29/109). Patients with HighT2 were more often at an intermediate-high risk of SCD according to the European (28 vs. 10%, p = .032) and American guidelines (41 vs. 18%, p = .010) compared to those without HighT2. The estimated 5-year SCD risk of our cohort was 1.9% (IQR 1.3–2.9%), and projected SCD rates were higher in patients with than without HighT2 (2.8 vs. 1.8%, p = .002). In conclusion, HCM patients with HighT2 were more likely to be intermediate-high risk, with projected SCD rates that were 1.5 fold higher than in patients without HighT2. These pilot findings call for corroborative studies with more intermediate-high risk HCM patients and clinical follow-up to assess whether HighT2 may have additional value to current risk stratification.

**Electronic supplementary material:**

The online version of this article (doi:10.1007/s10554-017-1252-6) contains supplementary material, which is available to authorized users.

## Introduction

Hypertrophic cardiomyopathy (HCM) is the most common inheritable cardiomyopathy and the most frequent cause of sudden cardiac death (SCD) among young athletes [1–3]. Unfortunately, identification of patients at risk of SCD remains challenging and new risk stratifiers such as biomarkers and imaging parameters are under investigation [4–8]. In this context, cardiovascular magnetic resonance (CMR) imaging is increasingly used for assessment of the extent of late gadolinium enhancement (LGE), as an indicator of fibrotic burden [6, 9, 10].

Another imaging feature with potential impact could be high signal intensity using T2-weighted CMR imaging (HighT2). This is based on the reported associations with markers of adverse disease progression, such as LGE, elevated troponin, and non-sustained ventricular tachycardia [5, 6, 11–13].

Whereas LGE is observed in about 60–70% of patients, HighT2 is observed in about one-third of HCM patients. Notably, areas of HighT2 are almost exclusively present in patients with LGE, occurring within the boundaries of LGE [13–20]. Appreciating that the prevalence of intermediate-high risk HCM patients is rather low, these specific characteristics of HighT2 may allow for refined stratification.

In addition, we have recently reported an independent association with an elevated level of cardiac troponin T, which supports that HighT2 is likely indicative of recently sustained myocyte injury [20]. In view of this, HighT2 may identify patients with a more active disease state, who might be vulnerable to adverse disease progression. The additional observations that HighT2 was associated with NSVT raised the question whether HighT2 might be a valuable predictor of adverse events, SCD in particular [14, 16, 18].

Importantly, studies with clinical follow-up on HighT2 are lacking. Therefore, we sought to provide the first pilot data on the association of HighT2 with the current SCD risk categorizations according to the ESC and ACC/AHA guidelines. We assessed the proportion of intermediate to high risk patients in relation to the presence or absence of HighT2. In addition, we performed an exploratory analysis on HighT2 and the associated projected SCD rates determined with use of the HCM Risk-SCD model, to provide insight into potential clinical implications [21].

## Methods

### Study population

For the present analysis, we studied a series of HCM patients who underwent T2-weighted CMR imaging, as participants of a Dutch multicentre HCM study project [20]. In short, enrollment took place between 2008 and 2014 at different outpatient clinics that perform mutation screening, repeated echocardiography, CMR imaging and clinical follow-up on a routine basis. Patients had to fulfill the diagnostic criteria for HCM according to the prevailing guidelines, which were assessed by a careful case-by-case chart review, especially in those with a history of hypertension [1, 2, 22]. Patients with known coronary disease or stroke, a history of out-of-hospital cardiac arrest, aortic stenosis, previous septal reduction therapy, renal impairment (MDRD < 30 ml/min) or a contraindication for CMR imaging were excluded. The study complies with the Declaration of Helsinki and the protocol was approved by the local ethical committees and conducted accordingly. All participants provided written informed consent.

### Cardiovascular magnetic resonance image acquisition and analysis

CMR imaging was performed on a 1.5T CMR system (Philips Achieva - Philips HealthCare, Best, The Netherlands or Siemens Avanto—Siemens Health Care, Erlangen, Germany) according to local imaging protocols, as previously described in more detail [20]. All images were acquired with ECG-gating and during repeated breath-holds of 10–15 s. To assess the presence of HighT2, breath-hold triple inversion-recovery T2-weighted images with fat-saturation were acquired (short-axis stack covering the left ventricle (LV) from base to apex). A long-axis image was obtained to exclude artifacts. For the assessment of LV function and mass, cine imaging was performed using a steady-state free precession sequence (short-axis stack covering the LV from base to apex). Segmented inversion-recovery imaging was performed to assess late gadolinium enhancement (LGE) 10 min after the administration of 0.2 mmol/kg contrast medium (Dotarem; Guerbet, Gorinchem, The Netherlands).

Images were analyzed with commercially available software (QMass 7.5, Medis, Leiden, The Netherlands) by two observers (FG and JB) unaware of the subjects’ clinical information. All 17 segments of the AHA-model were analyzed for the presence of HighT2 and LGE. HighT2 and LGE were scored visually per segment as either present or absent [15, 17]. In case of discrepancy between both observers on the presence of LGE or HighT2, a third observer (HD) reviewed the images for final adjudication. The observers were blinded for LGE data when analyzing T2-weighted images. The extent of LGE was determined according to a semi-quantitative score [23]. LV volumes, mass and ejection fraction were assessed using a standard protocol, as previously described [24, 25].

### Assessment of sudden cardiac death risk

For all participants, the following risk factors were recorded at the day of CMR imaging: age at evaluation; family history of SCD; history of unexplained syncope; NSVT on 24-h Holter monitoring; maximal LV wall thickness, LV outflow tract obstruction gradient (either resting or provocable gradient) and left atrial diameter measured using echocardiography; abnormal blood pressure response during exercise. Missing data was ~ 1%. For a detailed description and analysis of the risk factors, we refer to the appendix.

Our primary objective was to study the association between HighT2 and the categorization into low, intermediate or high risk of SCD according to the ESC and ACC/AHA guidelines [1, 2]. For the former, the HCM Risk-SCD calculator was used for estimation of the 5-year SCD risk, available at http://doc2do.com/hcm/webHCM.html. An intermediate risk was defined as an estimated 5-year SCD risk of ≥ 4–< 6% and a high risk as ≥ 6% [1, 26]. According to the ACC/AHA guidelines, patients were considered high risk in case of a family history of SCD, a history of unexplained syncope or extreme LV hypertrophy. In case of an abnormal blood pressure during exercise or NSVT on 24-h Holter monitoring, patients were recorded as intermediate or low risk depending on whether a risk modifier was present or not. Risk modifiers were a LV outflow tract gradient ≥ 30 mmHg or extensive LGE (≥ 15% of LV mass). Our secondary outcome measure was the estimated 5-year SCD risk as a continuous variable.

### Statistical analysis

Continuous variables are presented as means ± standard deviations or medians [interquartile ranges (IQR)], and were compared between patients with and without HighT2 using a Student’s *t* or Mann–Whitney *U* test, whichever appropriate. Dichotomous variables were compared using a Chi square or Fisher exact test, whichever appropriate. Given the previously reported co-localization of HighT2 with LGE [20], we also compared SCD risk in relation to LGE status, using a Kruskall-Wallis and Chi square test. A p value of < .05 was considered statistically significant. Statistical analysis was performed with IBM SPSS Statistics 20.0 (IBM Corp, Armonk, NY, USA).

## Results

### Study population

The present study population comprised of 109 HCM patients (56% male, age 54 ± 15), of whom the majority has previously been described [20]. 59 (58%) carried a pathogenic sarcomere mutation and atrial fibrillation was present in 18 (17%) patients (Table 1). Most patients were a- or mildly symptomatic with 105 (96%) patients in NYHA class I–II.

**Table 1:** Baseline characteristics of HCM patients with and without HighT2

	Total (n = 109)	HighT2 present (n = 29)	HighT2 absent (n = 80)	p value
Age (years)	54 ± 15	52 ± 14	55 ± 15	.29
Men	61 (56)	20 (69)	41 (51)	.10
Age at diagnosis (years)	47 ± 16	44 ± 15	49 ± 16	.20
Pathogenic mutation present	59 (58)	15 (58)	44 (58)	.99
Atrial fibrillation	18 (17)	5 (17)	13 (16)	1.0
Hypertension	40 (37)	9 (31)	31 (39)	.46
Symptoms				
Chest pain	21 (19)	3 (10)	18 (23)	.16
Dyspnea (NYHA class ≥ II)	49 (45)	17 (59)	32 (40)	.08
Therapy				
Beta-blocker	51 (47)	13 (45)	38 (48)	.81
Calciumantagonist	16 (15)	3 (10)	13 (16)	.55
Troponin T concentration (ng/L)	8 (3–14)	15 (8–25)	7 (3–12)	< .001
CMR Imaging				
LVMI (g/m^2^)	62 (52–87)	85 (63–116)	59 (51–74)	< .001
LV ejection fraction (%)	59 ± 7	55 ± 7	61 ± 6	< .001
LGE present (n)	68 (65)	26 (93)	42 (55)	< .001
LGE extent (% of LV mass)	3 (0–10)	10 (4–19)	1 (0–7)	< .001

Data are presented as means ± standard deviations, medians (interquartile ranges) or numbers (percentages)

*HighT2* high signal intensity on T2-weighted imaging, *NYHA* New York Heart association, *CMR* cardiovascular magnetic resonance, *LV* left ventricle, *LVMI* LV mass indexed to body surface area, *LGE* late gadolinium enhancement

**Fig. 1:**
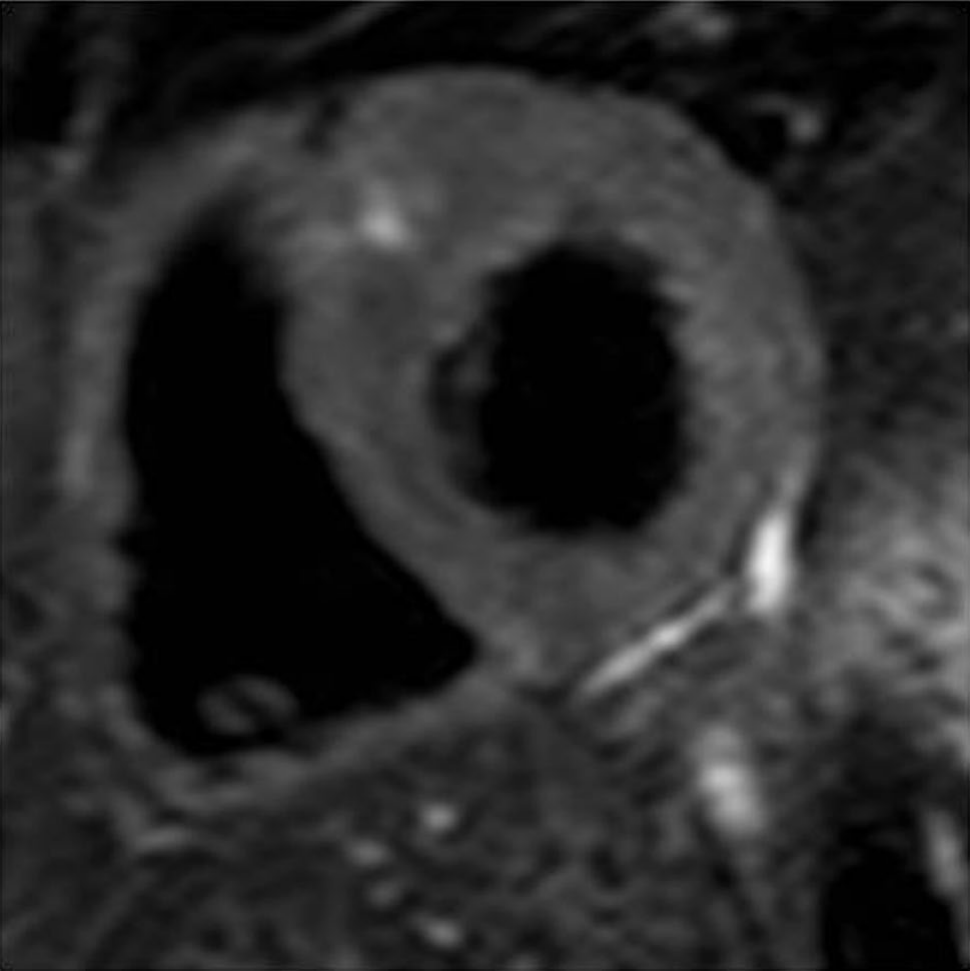
An imaging example of a HCM patient with HighT2. HighT2 was mostly demonstrated as a focal area in the hypertrophied anteroseptal wall at the insertion point of the right ventricle, as displayed here

Twenty-nine out of 109 (27%) were positive for HighT2. HighT2 was mostly observed midwall and co-localized within an area of LGE in hypertrophied segments, as previously described in more detail [20] (Fig. 1). In patients with HighT2, the median number of segments with HighT2 was 3 (IQR 2–4).

Patients with HighT2 tended to be more dyspnoeic and had a higher LV mass indexed to body surface area and a lower LV ejection fraction. Furthermore, cardiac troponin T concentrations were higher in patients with than without HighT2. The proportion of patients with LGE and the extent of LGE were also higher in the former group (Table 1).

### HighT2 and risk of sudden cardiac death

**Fig. 2:**
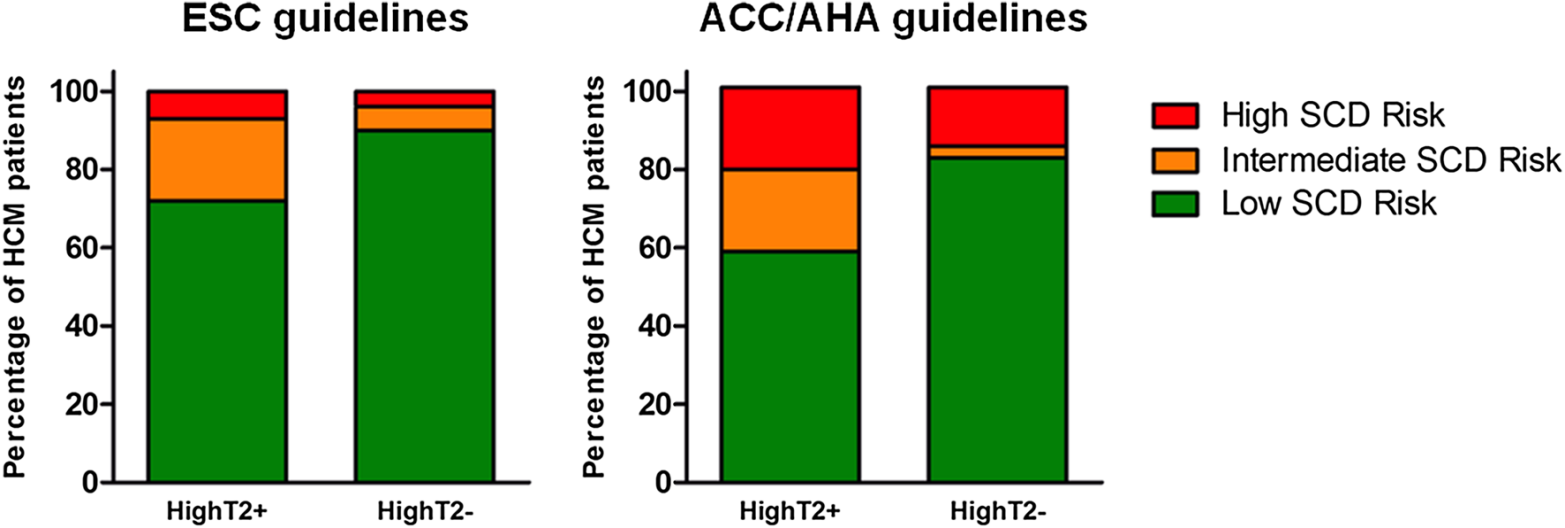
Risk categorization according to the ESC and ACC/AHA guidelines, stratified by the presence of HighT2. Left: according to the ESC guidelines, patients with HighT2 were more often at intermediate to high risk: 28% (8/29) versus 10% (8/80), p = .032. Of the 29 patients with HighT2, there were 21 at low risk of SCD; 6 and 2 were at intermediate and high risk, respectively. Of the 80 patients without HighT2, there were 72 at low risk of SCD; 5 and 3 were at intermediate and high risk, respectively. Right: according to the ACC/AHA guidelines, patients with HighT2 were more often at intermediate to high risk: 41% (12/29) versus 18% (14/80), p = .010. Of the 29 patients with HighT2, there were 17 at low risk of SCD; 6 and 6 were at intermediate and high risk, respectively. Of the 80 patients without HighT2, there were 66 at low risk of SCD; 2 and 12 were at intermediate and high risk, respectively

Patients with HighT2 were more often at an intermediate-high SCD risk according to the ESC guidelines (28 vs. 10%, p = .032) and ACC/AHA guidelines (41 vs. 18%, p = .010) (Table 2; Fig. 2). Projected mortality rates were higher in patients with HighT2, with a median estimated 5-year SCD risk of 2.8 versus 1.8% for patients without HighT2 (p = .002). The analysis on HighT2 combined with LGE status, demonstrated the lowest SCD risk in HCM patients without LGE and without HighT2. Moreover, in patients with LGE those with HighT2 had the highest SCD risk (Table 3).

**Table 2:** SCD risk profile in HCM patients with or without HighT2

	Total (n = 109)	HighT2 present (n = 29)	HighT2 absent (n = 80)	p value
Risk category				
ESC: Intermediate-high SCD risk	16 (15)	8 (28)	8 (10)	.032
ACC/AHA: Intermediate-high SCD risk	26 (24)	12 (41)	14 (18)	.010
Quantitative SCD risk				
Estimated 5-year risk (%)	1.9 (1.3–2.9)	2.8 (1.6–4.3)	1.8 (1.2–2.6)	.002

Data are presented as numbers (percentages) or medians (interquartile ranges)

*HighT2* high signal intensity on T2-weighted imaging, *SCD* sudden cardiac death

**Table 3:** SCD risk profile in HCM patients with or without HighT2/LGE

	LGE− and HighT2− (n = 35)	LGE+ and HighT2− (n = 42)	LGE+ and HighT2+ (n = 26)	p value
Risk category				
ESC: intermediate-high SCD risk	1 (3%)	7 (17%)	8 (31%)	.012
ACC/AHA: intermediate-high SCD risk	5 (17%)	9 (21%)	11 (42%)	.035
Quantitative SCD risk				
Estimated 5-year risk (%)	1.5 (1.1–1.9)	2.3 (1.4-3.0)	2.9 (1.6–4.3)	< .001

Data are presented as numbers (percentages) or medians (interquartile ranges)

*LGE* late gadolinium enhancement, *HighT2* high signal intensity on T2-weighted imaging, *SCD* sudden cardiac death

Table 4 displays the prevalence of each of the respective risk factors and risk modifiers stratified for the presence of HighT2. In addition to the significantly higher maximal wall thickness in patients with HighT2, the numerically higher proportion of NSVT and younger age contributed to the higher estimated 5-year SCD risk in these patients. With regard to the ACC/AHA risk model, extensive LGE was significantly more often present in patients with HighT2. Among HCM patients with an estimated low risk of SCD according to the ESC guidelines (n = 93), those without HighT2 had a significantly lower SCD risk score than those with HighT2 (1.7 vs. 2.0%, p = .021). Among those with an estimated high risk of SCD (n = 5), those without HighT2 (n = 3) had an estimated risk of SCD of 6.1, 6.2 and 6.9 vs. 7.4 and 9.6% in those with HighT2 (n = 2) (Supplementary Fig. 1).

**Table 4:** Individual risk factors and risk modifiers in HCM patients with or without HighT2

	Total (n = 109)	HighT2 present (n = 29)	HighT2 absent (n = 80)	p value
Binary risk factors				
Family history of SCD	12 (11)	4 (14)	8 (10)	.73
Syncope	5 (5)	2 (7)	3 (4)	.61
Non-sustained VT	17 (16)	6 (21)	11 (14)	.38
Abnormal BP response	13 (12)	7 (24)	6 (8)	.04
Extreme LV hypertrophy	3 (3)	1 (3)	2 (3)	1.0
Continuous risk factors				
Age (years)	54 ± 15	52 ± 14	55 ± 15	.29
Maximal wall thickness (mm)	17 (14–20)	19 (17–23)	16 (13–20)	< .001
Left atrial diameter (mm)	43 (39–50)	43 (40–54)	43 (39–48)	.26
LV outflow tract gradient (mmHg)	8 (6–23)	8 (5–21)	8 (6–25)	.74
Risk modifiers				
LV outflow tract gradient ≥ 30 mmHg	21 (19)	5 (17)	16 (20)	.75
Extensive LGE (≥ 15% of LV mass)	10 (9)	8 (28)	2 (3)	< .001

Data are presented as means ± standard deviations, medians (interquartile ranges) or numbers (percentages)

*HighT2* high signal intensity on T2-weighted imaging, *SCD* sudden cardiac death, *VT* ventricular tachycardia, *BP* blood pressure, *LV* left ventricle, *LGE* late gadolinium enhancement

## Discussion

This report represents a first exploratory analysis in the largest HCM cohort so far on the association between HighT2 and the risk categories for sudden cardiac death, as defined by the European and AHA/ACC guidelines.

Our pilot data demonstrate that HCM patients with HighT2 are more likely to be at intermediate to high risk of SCD, with projected SCD rates that are 1.5 fold higher than in patients without HighT2.

Importantly, apart from associations with some of the variables integrated in the HCM Risk-SCD model, HighT2 was also found to be related to markers of adverse disease progression not incorporated in the model (extensive LGE, LV mass and LV ejection fraction). When these findings are confirmed in larger cohorts with a higher proportion of intermediate-high risk patients, HighT2 may prove to be a valuable additive risk modifier or risk factor for future risk stratification schemes.

With the increasing use of CMR imaging in HCM, tissue characterization has become a topic of interest to further unravel pathophysiological aspects of the disease, and to determine the additional contribution of these imaging features in risk prediction of sudden death. From these studies we have learned that in almost all patients with HighT2 a substrate for arrhythmias was present in the form of fibrosis [13–20]. In addition, it has been demonstrated that the sympathetic tone is higher in patients with HighT2 [18]. These observations may explain why HighT2 has previously been associated with the occurrence of NSVT [14, 16, 20]. In addition, association with other markers of advanced disease have been reported [18, 20]. In this context, it has been hypothesized that HighT2 might be a predictor of adverse events, SCD in particular.

In our HCM population, patients with HighT2 were more often at an intermediate-high SCD risk, regardless of whether the European or Northern American guidelines were used. It is evident that in the large subset of our low risk HCM patients, projected SCD rates were significantly higher in patients with HighT2. Whether this can be extrapolated to intermediate-high risk patients remains to be determined.

On the one hand, it should be noted that this observed association may be confounded due to the association between HighT2 and some of the conventional risk factors [LV wall thickness in particular (Table 4)]. In addition, the predictive value of HighT2 does not seem to be high. However, it has repeatedly been demonstrated that the discriminative ability of the individual risk factors of the models is rather poor, and that it is the combination of factors that improves the predictive ability.

On the other hand, HighT2 was also associated with the risk modifier extensive LGE (ACC/AHA) and other indicators of disease severity such as higher LV mass, lower LV ejection fraction and higher troponin T concentration [5, 6, 9, 11, 27, 28]. These results suggest that HighT2 is not a mere surrogate marker of risk factors already included in the prevailing risk models, but might be a valuable composite marker of arrhythmic risk, that would otherwise remain concealed with the current risk stratification schemes. Notably, fibrosis may be a confounder for HighT2 in the prediction of SCD. However, among those with fibrosis, we have demonstrated that the patients with HighT2 were more often at an intermediate-high risk of SCD. These findings are supportive evidence to conduct larger studies on the potential impact of HighT2 in relation to LGE and the conventional risk factors.

### Potential impact of HighT2 on clinical practice

The observed 1.5 fold risk increase for SCD associated with HighT2 is in the same order of magnitude as observed for an extent of LGE of ≥ 15%, which is associated with an almost twofold increased risk [6, 9].

Based on our findings, several hypotheses for both low risk and intermediate-high risk patients could be addressed in future studies as potential implications. Our findings in the large group of low risk patients imply that T2-weighted CMR imaging might be able to increase the negative predictive value of current risk stratification schemes. At present, sudden cardiac death still occurs among low risk patients, and because of the high proportion of low risk patients in the general HCM population absolute numbers of cases with SCD are still considerable [29]. In the absence of HighT2, we may identify a subgroup of low risk patients, who are really at very low SCD risk. This could implicate that they could reliably be assured that SCD is highly unlikely to occur [30].

As for the impact of HighT2 in intermediate-high risk patients, the small sample size does not allow firm conclusions. Possibly, the presence or absence of HighT2 could help to differentiate between higher and lower risk patients, respectively. As for the former, we demonstrated associations with indicators of advanced disease, such as low ejection fraction and troponin. Moreover, our data suggest that HighT2 seems to differentiate among patients with LGE.

It should be acknowledged that approximately half of the patients at intermediate-high risk did not have HighT2. This may be interpreted as an undesirable “missing” of patients at a considerable estimated risk of SCD. However, it has been demonstrated in an independent validation cohort for the HCM-SCD risk model that with a SCD rate of around 5%, about 17 patients need an ICD implantation to prevent one SCD in 5 years [31]. In addition, it was demonstrated that, especially in high risk patients, the predicted SCD risk was higher than the observed SCD risk [31]. Consequently, in the majority of HCM patients at an estimated intermediate-high risk no SCD occurs and ICD implantation may preferentially have been avoided. It requires further study to investigate whether the absence of HighT2 in intermediate-high risk HCM patients may lower the odds of future SCD, and improve the number needed to treat to prevent one SCD.

### Future developments: an integrative CMR approach

In search of refinement of the current risk stratification models that are based on echocardiographic and clinical variables, we agree with European and Northern American experts that incorporating myocardial tissue characterization (LGE and HighT2) may be of additional value [32–35].

The positive predictive value of the mere presence of LGE proved to be limited by its prevalence of about 60–70% and the annual risk of SCD of only about 1% in a general HCM population. In response, the extent of LGE has become the topic of interest, with promising results in risk prediction [6, 9, 36]. Of interest, extensive LGE was more often present in patients with HighT2. However, more than half of the cases with HighT2 were observed in patients without extensive LGE. Inherently, there may be additional value for HighT2, which is also confirmed by our finding that among LGE positive patients those with HighT2 had the highest estimated SCD risk.

Previously, we have demonstrated that among HCM patients with LGE, those without HighT2 had the lowest troponin concentration, resembling that of patients without any LGE. Moreover, patients without HighT2 had a ninefold lower chance of extensive LGE. Whereas the number of intermediate-high risk patients is limited, our findings in the large cohort of low risk patients are more robust and imply that it is likely that the absence of HighT2 could improve the negative predictive value in these patients.

In summary, given the currently suboptimal risk stratification, the addition of HighT2 may be valuable to improve both negative and positive predictive values of the risk models.. Incorporation of the abovementioned CMR variables in (currently running) HCM follow-up studies may provide valuable information with regard to their independent association and additive predictive values [7, 8].

### Limitations

Although this is the largest cohort of HCM patients with T2-weighted imaging information, the main limitation of this study is the low risk profile of the study population. At the time of this study 5-year follow-up was available for 70% of our cohort. With projected 5-year follow-up rates of about 2%, we decided that clinical endpoints rather than estimated sudden death rates would not provide much additional value for the current study question.

In this context, we performed exploratory analyses on projected rates of SCD and our findings should therefore be considered as hypothesis generating, and do not validate HighT2 as a risk factor for SCD. Confirmative studies with clinical follow-up in a more intermediate-high risk population are warranted.

Furthermore, the technique under investigation is limited by a high signal-to-noise ratio and frequent artifacts. Unfortunately, T2-mapping sequences were not available at the start of our study, but these seem very promising and may lead to more objective data. In analogy to previous studies in HCM, the presence of HighT2 was visually assessed by two independent observers (FG and JB) [15, 17]. A third observer was required for final adjudication in 14 of 109 patients (Cohen’s kappa: 0.631, p < .001). Regardless of whether results of observer 1 or 2 were used, the differences in SCD risk between patients with and without HighT2 were consistent.

Lastly, we are well aware of the fact that the validity of the HCM Risk-SCD model has recently been challenged for Northern American patients [37]. Nonetheless, for the current analysis, the HCM Risk-SCD model has the advantage of quantification of projected risk of SCD.

## Conclusion

In an era where tissue characterization with CMR imaging has become topic of interest for SCD risk stratification, we are the first to demonstrate that HCM patients with HighT2 are more likely to be at intermediate to high risk of SCD, with projected SCD rates that are 1.5 fold higher than in patients without HighT2. Notably, HighT2 was not only associated with established risk factors, but also with several markers of a detrimental disease course, that are currently not incorporated in the HCM Risk-SCD model.

The present findings should be considered as “pilot data”, but do put forward the hypothesis that HighT2 might be valuable for future SCD risk stratification models in HCM.

## Electronic supplementary material

Below is the link to the electronic supplementary material.

Supplementary material 1 (DOCX 14 KB)

Supplementary material 2 (TIF 1108 KB)

Supplementary material 3 (DOCX 12 KB)
