# Supplementary material for: High T2-weighted signal intensity for risk prediction of sudden cardiac death in hypertrophic cardiomyopathy
Source: Int J Cardiovasc Imaging. 2017 Oct 23;34(1):113–20. doi: 10.1007/s10554-017-1252-6 (PMC5797557; doi:10.1007/s10554-017-1252-6)
Supplement: Supplementary file 1 — Supplementary material 1 (DOCX 14 KB) [file 10554_2017_1252_MOESM1_ESM.docx]

**APPENDIX: Detailed description of SCD risk factors**

**Definitions of risk factors for calculation of the estimated 5-year SCD risk according to the HCM Risk-SCD model**

*Age:* Age at evaluation.

*Family history of SCD:* History of sudden cardiac death in 1 or more first degree relatives under 40 years of age or SCD in a first degree relative with confirmed HCM at any age (post- or ante-mortem diagnosis).

*Unexplained syncope:* History of unexplained syncope at or prior to evaluation.

*Maximal wall thickness:* The greatest LV wall thickness using 2-D echocardiography at time of evaluation.

*Maximal left ventricular outflow tract gradient:* The maximum LV outflow gradient determined at rest or with Valsalva provocation (irrespective of concurrent medical treatment) using pulsed and continuous wave Doppler from the apical three- and five-chamber views. Peak outflow tract gradients were determined using the modified Bernoulli equation: Gradient= 4V^2^, where V is the peak aortic outflow velocity.

*Left atrial diameter:* Left atrial diameter determined by M-Mode or 2D echocardiography in the parasternal long axis plane.

*Non-sustained ventricular tachycardia: ≥*3 consecutive ventricular beats at a rate of ≥120 bpm and <30 s in duration on Holter monitoring (minimum duration 24 hours) at or prior to evaluation.

**Additional risk factors for categorization according to the AHA/ACC Guidelines**

*Extreme left ventricular hypertrophy:* LV wall thickness ≥ 30mm using 2-D echocardiography at time of evaluation.

*Abnormal blood pressure response during exercise:* The definition was met in case of any of the following: 1) Flat response: increase in systolic BP during the whole exercise period of <25mmHg compared with the resting systolic BP; or 2) Hypotensive response: Initial increase in systolic BP with a subsequent fall by peak exercise of >10mmHg from baseline or the peak BP.

**Missing data**

In summary, of all risk factor variables 11 out of 981 were missing (̴ 1%). In 9 cases there was no Holter monitoring available and the risk factor NSVT was considered negative. In one patient with a missing family history, it was considered negative. Finally, in one patient with a missing left atrial diameter, we used a value, that was normal for that patient’s sex based on current echocardiography guidelines.[1]

In case we would have considered the abovementioned dichotomous risk factors to be present/positive, it would have resulted in a different and higher risk category of 3 patients (of which 2 were HighT2 positive). This would not have affected the conclusion of our study.

The patient with a missing left atrial diameter was categorized as low risk. Even in case of an unlikely left atrial diameter of 67mm (the highest entry possible in the risk calculator), this would not have altered his risk category (<2% 5-year SCD risk). Consequently, this missing value has not influenced the conclusion of our study.

1. Lang RM, Badano LP, Mor-Avi V, Afilalo J, Armstrong A, Ernande L, et al. Recommendations for cardiac chamber quantification by echocardiography in adults: an update from the American Society of Echocardiography and the European Association of Cardiovascular Imaging. European heart journal cardiovascular Imaging. 2015;16:233-70.
