# Supplementary figures and images for: High T2-weighted signal intensity for risk prediction of sudden cardiac death in hypertrophic cardiomyopathy
Source: Int J Cardiovasc Imaging. 2017 Oct 23;34(1):113–20. doi: 10.1007/s10554-017-1252-6 (PMC5797557; doi:10.1007/s10554-017-1252-6)

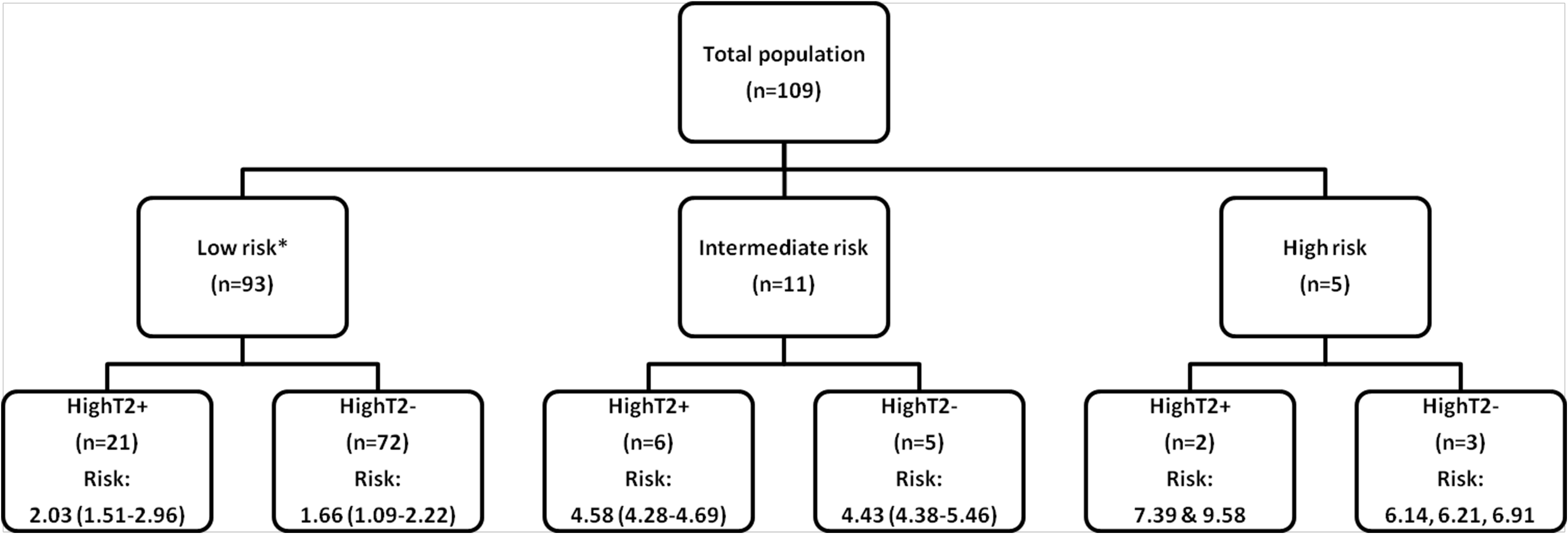

Supplement: Supplementary file 2 — Supplementary material 2 (TIF 1108 KB) [file 10554_2017_1252_MOESM2_ESM.tif]
