# Supplementary material for: High T2-weighted signal intensity for risk prediction of sudden cardiac death in hypertrophic cardiomyopathy
Source: Int J Cardiovasc Imaging. 2017 Oct 23;34(1):113–20. doi: 10.1007/s10554-017-1252-6 (PMC5797557; doi:10.1007/s10554-017-1252-6)
Supplement: Supplementary file 3 — Supplementary material 3 (DOCX 12 KB) [file 10554_2017_1252_MOESM3_ESM.docx]

|  | **MWT < median**  **(n=56)** | | **p** | **MWT > median**  **(n=53)** | | **p** |
| --- | --- | --- | --- | --- | --- | --- |
|  | **HighT2-**  **(n=49)** | **HighT2+**  **(n=7)** |  | **HighT2-**  **(n=31)** | **HighT2+**  **(n=22)** |  |
| **Risk category** |  |  |  |  |  |  |
| ESC: Intermediate-high SCD risk | 3 (6%) | 2 (29%) | .11 | 5 (16%) | 6 (27%) | .49 |
| ACC/AHA: Intermediate-high SCD risk | 8 (16%) | 4 (57%) | .03 | 6 (19%) | 8 (36%) | .21 |
| **Quantitative SCD risk** |  |  |  |  |  |  |
| Estimated 5-year risk (%) | 1.4 (1.1-2.0) | 2.2 (1.4-4.3) | .13 | 2.2 (1.7-2.9) | 2.9 (1.6-4.3) | .21 |

**Supplementary table 1. SCD risk profile in HCM patients wit hor without stratified according maximal wall thickness.**

Data are presented as numbers (percentages) or medians (interquartile ranges). HighT2 High signal intensity on T2-weighted imaging; SCD sudden cardiac death; MWT Maximal wall thickness.
